# Nurses’ Death Anxiety and Ageism towards Older Adults Amid the COVID-19 Pandemic: The Moderating Role of Symbolic Immortality

**DOI:** 10.3390/geriatrics7030063

**Published:** 2022-06-09

**Authors:** Mohammad Rababa, Shatha Al-Sabbah, Dania Bani-Hamad

**Affiliations:** Department of Adult Health Nursing, Faculty of Nursing, Jordan University of Science and Technology, Irbid 22110, Jordan; saalsabbah19@nur.just.edu.jo (S.A.-S.); debanyhamad19@nur.just.edu.jo (D.B.-H.)

**Keywords:** ageism, death anxiety, symbolic immortality, older adults, COVID-19, nurses

## Abstract

The coronavirus disease 2019 (COVID-19) pandemic has affected all aspects of individuals’ lives and behaviors, including the behaviors of nurses. Specifically, the pandemic has impacted the way that nurses treat older adults and has led to the spread of ageism among nurses. This study was conducted using self-report tools on 163 nurses to examine the problem of ageism amid the COVID-19 pandemic. The results suggest that critical care nurses have higher levels of death anxiety and ageism in comparison to medical/surgical nurses. After controlling for the work department, low levels of symbolic immortality were associated with high levels of ageism and death anxiety among nurses. These results might provide an insight into the development of a psychological intervention to reduce nurses’ death anxiety and ageism toward older adults.

## 1. Introduction

Ageism, which is defined as discrimination against older adults due to their age [1], is a highly prevalent issue in both Western and Eastern countries [2,3]. Nurses often hold many stereotypes about older adults, including the stereotype that older adults are difficult, frail, and incurable patients and that intensive nursing care, especially during disasters, should not be prioritized for older adults [3]. The COVID-19 pandemic has accentuated the exclusion of older adults from receiving mechanical ventilation support [4]. The outbreak of the disease has also highlighted the unfair allocation of healthcare resources and the mistreatment of older adults, raising questions regarding the extent to which the lives of older adults are valued by society [5]. Older adults are more susceptible to death from COVID-19 than other age groups [6]. Due to their high susceptibility to infection with COVID-19, older adults may be viewed by nurses as major transmitters of the disease and may thus be avoided [6].

The nursing literature has primarily focused on factors pertaining to negative attitudes towards older adults and knowledge of aging when exploring ageism among nurses [7,8]. However, nurses’ ageism towards older adults may be explained by their own underlying/disavowed death anxiety, which is defined as the unaware terror of one’s unavoidable mortality [9]. As hypothesized by terror management theory (TMT), older adults are viewed as a potent reminder of death, which may elicit nurses’ death anxiety and thus lead them to develop ageism in defense [10]. The COVID-19 pandemic has exacerbated the levels of death anxiety among nurses, and with older adults being the age group most susceptible to death from COVID-19, nurses may feel the urge to distance themselves from older adults [5].

**Figure 1:**
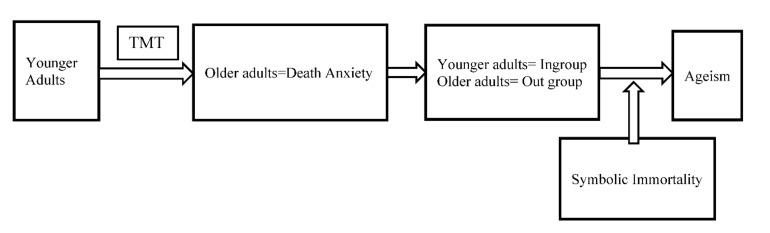
The etiological model for ageism among younger adults.

TMT also posits that individuals have an anxiety-buffering system called symbolic immortality [10], which refers to one’s sense of being a worthy element of reified individuals that are more enduring and significant than the individuals themselves [11,12]. Symbolic immortality plays a key role in informing people’s unconscious awareness of their mortality salience [13,14]. It is hypothesized that enhanced symbolic immortality may decrease nurses’ levels of death anxiety [10] and hence decrease their ageism towards older adults, and vice versa [2]. The etiological model for ageism among younger adults describes these associations based on the TMT (Figure 1).

Recent studies have evidenced the presence of a positive association between death anxiety and ageism [15] and a negative association between symbolic immortality and ageism [16]. However, these studies have not considered the moderating (buffering) effect of symbolic immortality on the relationship between death anxiety and ageism among nurses. In other words, no previous study has explored how the association between nurses’ death anxiety and ageism depends on their levels of symbolic immortality. In the current study, we hypothesized that the relationship between nurses’ death anxiety and ageism is moderated by their levels of symbolic immortality. Moreover, no studies to date have examined the associations of the nurses’ demographic (i.e., gender and marital status) and professional (i.e., level of education, work department, and years of experience) characteristics with their symbolic immortality, ageism, and death anxiety. Although recent studies have partially evidenced an association of ageism and death anxiety with age, gender, and marital status [17,18], these studies have not considered the interaction between these demographic variables and symbolic immortality among nurses. The current study aimed to examine (1) the differences in symbolic immortality, death anxiety, and ageism against older adults between the nurse groups according to certain sociodemographic and professional characteristics and (2) the moderating effect of symbolic immortality on the relationship between death anxiety and ageism in nurses caring for older adults. Hence, the current study was guided by the following research question and hypothesis.

## 2. Research Question

Are there differences in the levels of symbolic immortality, death anxiety, and ageism against older adults between the nurse groups according to certain sociodemographic (i.e., marital status and gender) and professional (i.e., work department, years of experience, level of education) characteristics?

## 3. Research Hypothesis

Controlling for professional and sociodemographic characteristics, nurses’ levels of symbolic immortality moderate the relationship between their death anxiety and ageism towards older adults.

## 4. Methods

### 4.1. Design and Sample

This cross-sectional study was conducted between April and May 2021 in a government hospital. One hundred and sixty-three nurses with clinical experience of at least 12 months were conveniently recruited in this study. G-power analysis for Multivariate analysis of variance (MANOVA) was used to calculate the required sample size [19]. A minimum of 143 nurses was required in this study, giving an estimated medium effect size of 0.06, a desired statistical power level of 0.95, an alpha level of 0.05, three comparison groups, and three response variables. To account for any potential dropouts, 20 additional nurses were recruited, with a final total sample of 163 nurses.

### 4.2. Study Measures

Ageism. Nurses’ levels of ageism were measured using the Fraboni Scale of Ageism [20]. The FSA is a 29-item scale, measuring the affective and attitudinal components of ageism. The items of the FSA are graded on a 4-point Likert scale ranging from “1 = strongly disagree” to “4 = strongly agree.” The FSA has three positive items (e.g., “Old people should feel welcome at the social gatherings of young people”), which were reverse coded prior to calculating the total score. The total possible score of the FSA ranges from 29 to 116, with a higher score representing a greater level of ageism. The Cronbach’s alpha reliability of the FSA in the current study was high (0.89).

Death anxiety. Nurses’ levels of death anxiety in the current study were measured using the revised Collett–Lester Fear of Death Scale [21]. The CL-FODS has four subscales, namely, (1) individual’s own death; (2) individual’s own dying; (3) the dying of others; and (4) death of others, with seven items for each subscale. The CL-FODS items are rated on a 5-point Likert scale ranging from 1 = “no” to 5 = “very”. The total possible score of the CL-FODS ranges from 28 to 140, with higher scores representing increased levels of death anxiety. The Cronbach’s alpha reliability of the CL-FODS in the current study was satisfactory (0.79).

Symbolic immortality. The 26-item Sense of Symbolic Immortality Scale (SSIS) was used in the present study to measure nurses’ levels of symbolic immortality [22]. The SSIS includes 11 negative items (e.g., “I am of no value in the eyes of society”), which were reverse coded prior to calculating the total score. The SSIS items are rated on a 5-point Likert scale ranging from “1 = strongly disagree” to “5 = strongly agree”. The total possible score ranges from 26 to 130, with a higher score indicating a greater sense of symbolic immortality. The Cronbach’s alpha reliability of the SSIS in the current study was very good (0.86).

Demographic data. A demographics questionnaire developed by the researcher was utilized to collect data about some selected sociodemographic and professional characteristics of the nurses. The sociodemographic characteristics included gender (i.e., male vs. female.), age, and marital status (i.e., single vs. married.). The professional characteristics included level of education (i.e., BSN vs. MSN), level of clinical experience (i.e., senior “> 5 years of clinical experience” vs. junior “≤ 5 years”), and work department (i.e., medical/surgical, ICCU/CCU, or ER).

### 4.3. Data Collection

Institutional ethics approval was granted by the Institutional Review Board department at the researcher’s university (IRB number = 267-2018). After that, the researcher met with the administrative personnel of the targeted hospital to discuss the inclusion criteria of the study and obtain a list of potential participating nurses’ names and emails. An invitation email containing brief information about the study purposes and procedure was sent via the hospital’s email portal. All invited nurses who agreed to participate in the study provided written informed consent. Each nurse who agreed to participate received a pack including all of the study questionnaires, and the nurses were asked to turn in their folders to the manager’s office upon completion of the questionnaires.

### 4.4. Data Analysis

Data analysis was conducted using the Statistical Package for the Social Sciences version 25.0 (IBM Corp, Armonk, NY, USA). To determine the statistical significance of the statistical tests used, a level of significance of α = 0.05 was used. Descriptive analysis was used to analyze the nurses’ professional and sociodemographic characteristics and their levels of ageism, death anxiety, and symbolic immortality.

Given the conceptual/theoretical interconnectedness of the study variables, one-way MANOVAs and subsequent individual ANOVAs with the Bonferroni post hoc test were applied to examine the differences in the study variables between the nurse groups. All assumptions of multivariate and univariate normality, homogeneity of variances, and normal distribution were checked and met. SPSS PROCESS macro (version 3.5; Model 1; [23]) was utilized to examine the moderating effect of symbolic immortality in the current study. Before conducting the moderation analysis, mean centering of the predictor and moderator variable scores were used to avoid multicollinearity. Subsequently, the moderation analyses were followed by models controlling for only the significant sociodemographic and professional variables based on the results of the MANOVAs to evaluate the extent to which symbolic immortality’s influence was independent of these control variables.

## 5. Results

### 5.1. Participants’ Demographic Characteristics

The sample consisted of 163 nurses with an average age of 30.47 years (SD = 5.04). The majority of the nurses were female (54.4%), married (64.4%), senior (50.3%), holders of a bachelor’s degree in nursing (73.6%), and working in the medical/surgical department (50.3%). The descriptive statistics of the nurses’ sociodemographic and professional characteristics are outlined in Table 1.

### 5.2. Description of the Nurses’ Mean Scores

As is evident from Table 1, the participating nurses had an above-average level of symbolic immortality (M = 97.27, SD = 21.52). Meanwhile, the participating nurses had high levels of death anxiety (M = 96.93, SD = 23.69) and ageism (M = 83.09, SD = 20.21).

### 5.3. Comparisons Based on the Selected Sociodemographic/Professional Characteristics

The overall MANOVA results indicated statistically significant differences between the nurse groups based on work department: [F (3, 158) = 2.47, *p* = 0.013, partial η^2^ = 0.059]. This finding suggested that there were significant differences in scale scores between ICU/CCU, ER, and medical/surgical nurses. Subsequent individual ANOVAs were performed to further examine the differences in symbolic immortality, death anxiety, and ageism scores. The results of the individual ANOVAs were statistically significant for the level of symbolic immortality [F (2, 160) = 7.23, *p* = 0.001, partial η^2^ = 0.083], death anxiety [F (2, 160) = 3.98, *p* = 0.021, partial η^2^ = 0.047], and ageism [F (2, 160) = 3.41, *p* = 0.036, partial η^2^ = 0.041]. ICU/CCU nurses reported lower levels of symbolic immortality and higher levels of death anxiety and ageism than did medical/surgical nurses and ER nurses. Table 2 presents the results of the overall MANOVAs and the individual ANOVAs of the differences between the nurses grouped according to their work department. The overall MANOVA results indicated no statistically significant differences between the nurse groups based on the other sociodemographic and professional variables.

### 5.4. Predictors of Nurses’ Ageism

**Figure 2:**
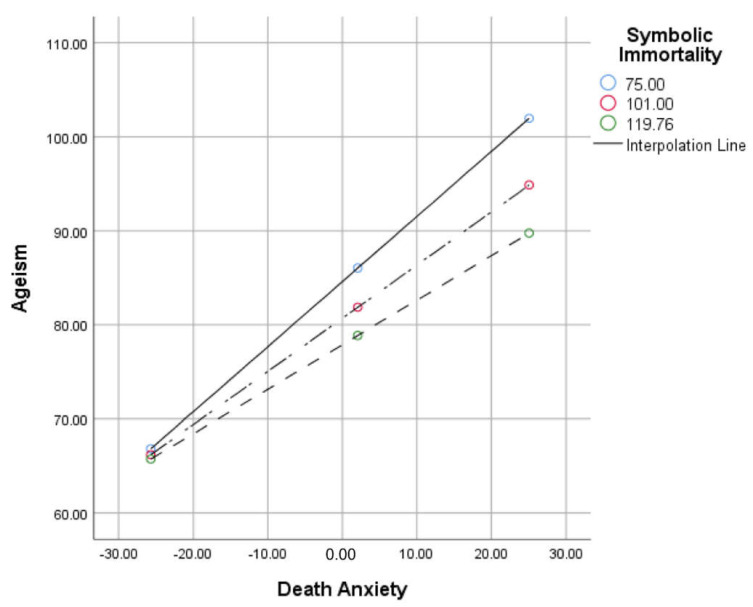
The relationship between death anxiety and symbolic immortality at low, moderate, and high levels. Y axis exhibits total score on Ageism. X axis exhibits mean-centered CL–FODS scores.

A moderation analysis examined whether the relationship between death anxiety and ageism was moderated by symbolic immortality. The overall model was significant, F (3, 159) = 90.86, *p* < 0.001, R^2^ = 0.63. Death anxiety and symbolic immortality were significant predictors of ageism. Symbolic immortality moderated the relationship between death anxiety and ageism, ΔR^2^ = 0.12, F = 5.14, *p* = 0.025, β = −0.005. An additional model controlling for the work department remained significant, F (5, 157) = 54.42, *p* < 0.001, R^2^ = 0.63. Symbolic immortality remained a significant moderator of the relationship between ageism and death anxiety, ΔR^2^ = 0.01, F = 5.01, *p* = 0.027, β = −0.005. Figure 2 shows a graphical illustration of symbolic immortality moderating death anxiety’s relation to ageism. The model also revealed that the work department did not significantly predict nurses’ levels of ageism independently. Please see Table 3. 

## 6. Discussion

This study is the first to examine death anxiety and symbolic immortality and how they related to ageism towards older adults among nurses during the COVID-19 pandemic. This study found significant differences in death anxiety and ageism between the nurse groups based on work department. Furthermore, our findings supported the main hypothesis that the nurses’ levels of death anxiety and symbolic immortality would predict their levels of ageism. Further, consistent with TMT, symbolic immortality was found to have a significant moderating effect on the relationship between nurses’ death anxiety and ageism towards older adults.

Consistent with findings reported by previous studies regarding ageism [8] and death anxiety [24], the current study found levels of death anxiety and ageism to be high among the participating nurses. Previous studies have found that providing care for older adults amid the COVID-19 pandemic may trigger nurses’ sense of death anxiety, leading them to avoid emotional engagement with older adults and socially distance themselves from them [5]. In order to cope with their death anxiety, they may adopt ageist behaviors, such as elder mistreatment and neglect as nontherapeutic coping strategies [25].

In the current study, the nurses’ mean SISS score was higher than that reported in previous studies [22,26,27]. This relatively high level of symbolic immortality among the participating nurses may be attributed to the high level of cultural observance and religiousness among Middle Eastern populations. Middle Eastern cultural values hold that individuals’ lives are meaningful in the afterlife as well as in this life [28]. Moreover, it is the teaching of Islamic tradition that life is temporary and will be followed by an afterlife in which humans will enjoy immortal existence [29,30]. These religious and cultural beliefs may often function as defense mechanisms making people believe that symbolic immortality is more long-lasting than physical existence [28]. Furthermore, Middle Eastern culture highly values strong family structure and bonds and emphasizes the importance of close ties between family members [31]. Future research is recommended to qualitatively examine the intercultural conceptualizations of symbolic immortality.

The significant results of the one-way MANOVAs highlighted the individual differences in the nurses’ levels of death anxiety and associated ageism based on their work departments. ICU/CCU nurses had significantly higher levels of death anxiety and ageism than did medical/surgical nurses, potentially indicating that death-related issues that increase death anxiety and its associated ageism occur more frequently in critical care settings than on regular floors [8]. During times such as the COVID-19 pandemic, ICC/CCU nurses may be more exposed than surgical/medical nurses to these death-related issues and their psychological effects, especially given that most COVID-19 patients admitted to ICUs/CCUs are critically ill or dying older adults. Meanwhile, surgical/medical nurses are often responsible for caring for stable cases of COVID-19 [16].

This finding that ICC/CCU nurses experience higher levels of ageism and death anxiety toward older adults is consistent with TMT and with previous studies [5,32]. About 65% of all COVID-19 confirmed cases have been among older adults, who have higher COVID-19-related mortality rates than other age categories [32]. Therefore, older adults with COVID-19 need to be admitted to critical care units for mechanical ventilation support more often than any other age group, which may lead ICU/CCU nurses to encounter more death-related stressors and experience higher levels of death anxiety than surgical/medical nurses. Due to the high vulnerability of older adults to COVID-19 and due to social distancing policies being aimed mainly at older adults, ICC/CCU nurses have been found to avoid socializing with older adults [5]. Further, with the rapid increase in COVID-19 cases requiring mechanical ventilation support being met with the limited number of mechanical ventilators, ICC/CCU nurses may feel that these scarce health care resources should be prioritized for younger adults [4].

The finding that death anxiety and symbolic immortality are significant predictors of ageism towards older adults is consistent with TMT and previous studies [33]. Earlier research studies have indicated that dealing with older adults may elicit a sense of death anxiety [33], thus leading to the avoidance and neglect of older people [15]. It has also been found that when an individual who has a low level of symbolic immortality is unconsciously elicited by mortality salience, they symbolically link death with older adults, as if they are a strong reminder of mortality [29,34]. Although this is the first study to explore the moderating effect of symbolic immortality, the finding is consistent with TMT [10]. According to TMT, nurses having high levels of symbolic immortality are less likely than nurses having low levels of symbolic immortality to hold ageist attitudes towards older adults [16]. It is hypothesized that symbolic immortality has a buffering effect on death anxiety and hence on ageism. Therefore, enhancing nurses’ symbolic immortality in the workplace may decrease their levels of death anxiety and ageism toward older adults [16]. It is crucial to enhance nurses’ engagement in charitable activities targeting older adults to allow nurses to better understand the meaning of this life and the afterlife and hence confront their mortality salience and manage their death anxiety. Further, during the COVID-19 pandemic, online applications such as Zoom or Skype can be used to increase nurses’ interaction with older adults. Enhanced intergenerational contact has been approved as an effective way of relieving ageism [31].

### 6.1. Implications for Research and Practice

The current study has several implications for future research and practice. A better understanding of the role of symbolic immortality may allow hospital administrators and health policymakers to develop ongoing training or educational programs that target death anxiety and its associated ageism among nurses. Furthermore, in order to enhance nurses’ symbolic immortality and thus improve their death anxiety and ageism, the implementation of psychological interventions, such as cognitive-behavioral therapy, is recommended [35]. Several research studies have examined the effectiveness of certain cognitive behavioral therapy exercises in enhancing the anxiety buffering system by improving symbolic immortality [36]. Moreover, intergeneration contact would benefit in relieving ageism by improving nurses’ knowledge of aging and attitudes toward older adults [31].

### 6.2. Study Limitations

Despite the novelty of the study findings, the study had several limitations. First, the descriptive, cross-sectional design used in this study did not allow for causal inference. Therefore, longitudinal studies are recommended in the future to further validate the study findings. Second, the use of self-report tools may have led to recall bias, which may threaten the reliability of the collected data. Third, the convenience sampling method is associated with selection bias, which may threaten the internal validity of the findings. Fourth, the participating nurses’ pre-pandemic levels of ageism, death anxiety, and symbolic immortality were not measured. Finally, the work is related to the Middle Eastern culture and the Islamic religion because this study was conducted in Jordan, which is an Islamic country, but other religions such as Christianity, Judaism, and Hinduism would also have a similar impact. Therefore, the study needs to be replicated in other countries with different religions to enhance the generalizability of the findings of the current study. 

## 7. Conclusions

The present study confirmed the significant moderating effect of symbolic immortality on the relationship between nurses’ levels of death anxiety and ageism towards older adults amid the COVID-19 pandemic. Therefore, future research studies are highly recommended to examine the effectiveness of educational, psychological, and spiritual interventions in improving nurses’ symbolic immortality and their understanding of death and dying, thus in relieving nurses’ death anxiety and ageism toward older adults.

## Figures and Tables

**Table 1:** Participant demographic characteristics (*n* = 163).

Sociodemographic and Professional Characteristics	*n*	%
Gender		
*Female*	*89*	*54.4*
*Male*	*74*	*45.4*
Marital Status		
*Single*	58	35.6
*Married*	105	64.4
Level of Education		
*Bachelor of Science in Nursing*	120	73.6
*Master of Science in Nursing*	*43*	*26.4*
Level of Experience		
*Senior (> 5 years of clinical experience)*	*82*	*50.3*
*Junior (≤ 5 years of clinical experience)*	*81*	*49.7*
Working Department		
*Medical/Surgical*	*82*	*50.3*
*Intensive Care Unit/Critical Care Unit*	*46*	*28.2*
*Emergency Room*	*35*	*21.5*
	**Mean**	**SD**
Age	30.47	5.04
Symbolic immortality	97.27	21.52
Death anxiety	96.93	23.69
Ageism	83.09	20.21

**Table 2:** One-way univariate and multivariate ANOVA of the study variables of the nurse groups (*n* = 163).

Variables	Work Department				
	Med/Surg M (SD)	ICU/CCU M (SD)	ER	F	Partial η^2^
Combined effect ^a^				2.5 *	0.06
Symbolic immortality	105.6 (23.3)	88.4 (22.6)	98.7 (18.3)	7.2 *	0.08
Death anxiety	87.7 (20.9)	102.2 (23.2)	97.9 (24.2)	4.0 *	0.05
Ageism	77.2 (14.7)	88.7 (17.7)	82.5 (22.7)	3.4 *	0.04

^a^: MANOVA; ANOVA: analysis of Variances; SD: standard deviation; M: mean; Med/Surg: Medical/Surgical floors; ER: Emergency Room; ICC/CCU: Intensive Care Unit/Critical Care Unit, * *p* < 0.05.

**Table 3:** Predictor and interaction statistics (*n* = 163).

Variable	β	s.e.	*t*	*p*	(95% Cl)
**Model 1**					
Death anxiety (centered)	1.040	0.228	4.553	<0.001	[0.5889, 1.4911]
Symbolic immortality	−0.161	0.067	−2.389	0.018	[−0.2942, −0.0279]
Death anxiety–symbolic immortality interaction	−0.005	0.002	−2.266	0.025	[−0.0091, −0.0006]
**Model 2**					
Death anxiety (centered)	1.058	0.232	4.545	<0.001	[0.5986, 1.5187]
Symbolic immortality	−0.150	0.069	−2.190	0.030	[−0.2853, −0.0147]
Death anxiety–symbolic immortality interaction	−0.005	0.002	−2.238	0.027	[−0.0092, −0.0006]
Work department	−1.115	1.996	−0.559	0.577	[−5.0588, 2.8282]

## Data Availability

The data presented in this study are available on request from the corresponding author. The data are not publicly available due to the privacy and confidentiality of the data.
